# The chemical blueprint of *Trichoderma* for next-generation microbial biostimulants

**DOI:** 10.1007/s11306-026-02531-4

**Published:** 2026-10-03

**Authors:** Thuso Mudau, Lerato Pertunia Tshehlane, Keabetswe Tebogo Ncube, Fidele Tugizimana

**Affiliations:** 1 Research Centre for Plant Metabolomics, Department of Biochemistry, University of Johannesburg, Auckland Park, Johannesburg, South Africa; 2 International Research and Development Division, Omnia Group, Ltd., Bryanston, Johannesburg, 2021 South Africa

**Keywords:** *Trichoderma* strains, *Bacillus* consortium, Metabolomics, In vitro assays

## Abstract

**Introduction:**

Microbial biostimulants comprise beneficial microorganisms, such as *Bacillus* and *Trichoderma*, that enhance plant growth, nutrient uptake, and stress tolerance through biological mechanisms. The combined use of *Bacillus* and *Trichoderma* is increasingly recognised as an important strategy for enhanced crop productivity and sustainable agriculture. However, the compatibility of microorganisms is constrained by their distinct ecological niche and metabolic activities, which may limit the stability of consortia.

**Objectives:**

To characterize the biochemical landscape of *Trichoderma* strains and evaluate their metabolic compatibility with the *Bacillus* consortium.

**Methods:**

Metabolites were extracted from each *Trichoderma* strain using LC–MS grade quality methanol. The methanolic extracts were analysed on a UPLC–MS system. Principal component analysis and orthogonal partial least squares discriminant analysis were performed to assess sample clustering and overall metabolic variation among *Trichoderma* strains. Feature-based molecular networking was used for metabolite annotation of *Trichoderma*’s chemical space. Functional analysis was used to map annotated metabolites in the metabolic pathways of *Trichoderma* strains.

**Results:**

The findings revealed distinct metabolic markers, including primary and secondary metabolites. The sphingolipid metabolism was significantly impacted, among others. The in vitro studies revealed a positive interaction with no evidence of antagonism between *Trichoderma* strains and the *Bacillus* consortium.

**Conclusion:**

The major constraint of biostimulant application is the lack of standardisation in product composition. Therefore, these findings provide insights into the molecular composition and functional dynamics of microbial networks. Such knowledge is crucial and essential for the development of cutting-edge innovations in the field of biostimulants.

**Supplementary Information:**

The online version contains supplementary material available at https://doi.org/10.1007/s11306-026-02531-4.

## Introduction

Sustainable agriculture focuses on achieving high yields without causing land degradation or damaging the resources that productivity depends on. The use of agriculturally important microorganisms, namely beneficial fungi (e.g., *Trichoderma)* and plant growth-promoting rhizobacteria (e.g., *Bacillus*), brings stability in agricultural systems (Hatami & Ahangarani, 2016; Pretty & Bharucha, 2014). Furthermore, the co-inoculation of *Trichoderma* with *Bacillus* may facilitate a synergistic interaction that enhances microbial activity and functional performance. Thus, improving nutrient solubilisation, microbial persistence, and suppresses pathogenic microorganisms compared with single inoculations (Aamir et al., 2026). For instance, the co-inoculation of *T. harzianum* and *B. flexus* was tested through in vitro screening, and results revealed no inhibitory effects of growth between *B. flexus* and *T. harzianum*, thereby ensuring compatibility between them (Singh et al., 2019). Therefore, successful co-formulation depends on microbial viability, environmental conditions, and formulation stability (Herrero et al., 2025). These factors are essential to maintain viability and minimise antagonistic interactions during storage and application (Sadanov et al., 2026; Sethi et al., 2025). However, significant knowledge remains to be addressed. At the formulation level, maintaining microbial viability during storage, transport, and field application is hindered by limited shelf life and inadequate carrier technologies, both critical to ensuring stability and consistent performance (Khan et al., 2023; Monica et al., 2025; Parveen et al., 2023). Scaling up production remains challenging, as transitioning from laboratory success to cost‑efficient industrial-scale fermentation and formulation demands robust quality control systems to safeguard cell viability, purity, and accurate labelling (Díaz-Rodríguez et al., 2025). In practice, field efficacy often varies widely due to interactions with native soil microbiota, environmental factors, and crop-specific conditions, reducing reliability across diverse agroecosystems (Herrmann & Lesueur, 2013). Compounding these issues are regulatory constraints, such as restrictive approvals, outdated frameworks, and insufficient farmer awareness, all of which inhibit adoption and market growth (Khoulati et al., 2025)

This study involves the use of metabolomics approaches for a comprehensive characterisation of the *Trichoderma* metabolome, revealing metabolites and signalling molecules that define its metabolic functionality and ecological interactions (Silva et al., 2024). Integrating metabolomic profiles of *Trichoderma* with its functional characteristics offers key insights into the biochemical pathways, molecular mechanisms, and chemical signalling processes that drive its behaviour and effectiveness. Thereby guiding its optimisation and application in microbial formulations (Mashabela et al., 2022; Shahriar et al., 2022; Thakur et al., 2025). Furthermore, the in vitro assay was employed to evaluate the interaction between *Bacillus* and *Trichoderma* under controlled conditions. These assist with determining whether the strains are compatible, antagonistic, or neutral before co-formulation (Sadia et al., 2026). Thus, reducing variability, allowing early detection of inhibition, and supporting the selection of suitable microbial combinations (Vasseur-Coronado et al., 2021). Accordingly, the distinctive contribution of this study is the integration of comparative untargeted metabolomic profiling of *T. asperellum* and *T. harzianum* with experimental assessment of their compatibility with a defined *Bacillus* consortium. This approach enables the identification of species-associated chemical signatures and metabolic pathways while simultaneously evaluating the suitability of these fungal systems for microbial co-formulation. The study therefore provides a chemical and compatibility framework to support the rational selection of microorganisms for multi-microbial biostimulant development.

## Materials and methods

The organic solvent used, methanol, was LC–MS grade quality (Romil, SPS, Cambridge, United Kingdom). Two powdered fungal biomass materials, *Trichoderma asperellum* and *Trichoderma harzianum*, were supplied by the Council for Scientific and Industrial Research (CSIR), South Africa, and are hereafter referred to as TA and TH, respectively. These materials were not isolated by the authors. Strain/accession numbers were not provided with the supplied materials. The powdered materials consisted of fungal biomass and did not contain formulation carriers or other excipients. The materials were not cultured by the authors prior to metabolite extraction, and the specific upstream culture conditions used for biomass production were not available to the authors. Following receipt, the powdered fungal biomass was stored at room temperature under dry conditions until metabolite extraction. The *Trichoderma*-based materials are currently under development for potential commercialisation, and additional proprietary information relating to their development cannot be disclosed due to ongoing research and intellectual property considerations. The *Bacillus* consortium is a commercial product, known as Bacstim®100 (Omnia Group Ltd., South Africa), containing equal volumes of *B. licheniformis M017*, *B. licheniformis 1001*, *B. amyloliquefaciens*, and *B. laterosporus* strains; and this will be referred to as B, for simplified semantics.

### Metabolite extraction

One gram (1 g) of powdered fungal biomass from each *Trichoderma* species (*T. asperellum* and *T. harzianum*) was transferred into separate microcentrifuge tubes, followed by the addition of 1.5 mL of 100% LC–MS grade methanol (Romil, SPS, Cambridge, United Kingdom). Methanol was selected because it is widely used as a single extraction solvent in metabolomics, providing efficient protein precipitation and rapid quenching of enzymatic activity while extracting a broad range of polar and semi-polar metabolites. Although broader metabolome coverage can be achieved using complementary solvents or biphasic extraction systems, the methanol-based extraction was considered appropriate for the comparative untargeted metabolomic profiling performed in the present study. The suspensions were vortexed and sonicated for 60 s using a probe sonicator (Bandelin Sonopuls, Germany) to break open the fungal cells. The mixtures were then centrifuged at 13,000 ×* g* for 15 min. Nine independent biological replicates were prepared for each *Trichoderma* strain, and the resulting supernatants were individually filtered into glass vials fitted with 500 µL inserts using 0.5 mL syringes and 0.22 µm nylon filters. Analytical blank samples were included during LC–MS/MS analysis to identify background signals and potential contaminants introduced during sample preparation and analysis. Pooled quality control (QC) samples were prepared by adding an equal volume of supernatant from each sample. Following extraction and filtration, the methanolic extracts were maintained at 4 °C for a short period prior to LC–MS/MS analysis.

### Data acquisition: LC–MS/MS

An ultra-performance liquid chromatography (UPLC) system, coupled in series to a SYNAPT XS mass spectrometer (Waters Corporation, Milford, MA, USA), was used to generate mass spectral data. The chromatographic separation was performed using a reverse-phase ACQUITY UPLC® HSS T3 C18 μm column (150 mm × 2.1 mm, 1.8 μm) (Waters), at a temperature of 60 °C. A binary solvent mixture was used, consisting of water (solvent A) containing 10 mM formic acid (pH of 2.4) and acetonitrile (solvent B) containing 10 mM formic acid. The gradient elution was performed as follows: Solvent B, referring to organic composition: 10% B for 1.00 min, a gradual increase up to 90% B over 1–15.00 min, 90–99% B over 15–15.10 min, and was kept constant until 17.00 min. The gradient was then changed back to 90% B at 17.10 min and kept for 3.90 min. The total runtime was 21 min with an injection volume of 2 μL. For mass spectrometry (MS) analyses, ionisation was carried out in both electrospray ionisation positive and negative modes with 100–1500 Da and 0.1 s scan range and scan time, respectively. The positive- and negative-ionization datasets were evaluated separately and were not merged. Preliminary evaluation indicated that the ESI-positive mode provided good analytical performance, including well-resolved chromatographic features, clear ion peaks, and suitable MS/MS spectral information for metabolite annotation. Therefore, the ESI-positive dataset was selected for the subsequent metabolomic analyses and is reported in the present study. Other MS parameters were set as follows: source temperature of 120 °C; leucine enkephalin (50 pg/mL) was used as reference calibrant (lock mass) to obtain typical mass accuracies between 1 and 5 mDa. The mass spectrometer was operated with a capillary voltage of 0.6 kV and the sampling cone at 30 V. A data-independent acquisition (DIA) method was applied (with collision energy ramping from 20 to 40 eV) to obtain fragmentation spectral data. All study samples were analysed within a single LC–MS/MS analytical batch. Pooled QC samples were injected at the beginning, middle, and end of the analytical sequence to monitor instrument performance and potential signal drift throughout the run.

### Data processing and multivariate data analysis

Following preliminary evaluation of the separately acquired ionization-mode datasets, the selected ESI-positive mass spectral data files (.raw, Waters) were converted to.mzML files using MS Converter software. The.mzML files were uploaded to the Mass Spectrometry Data Independent AnaLysis software (MS-DIAL version 4.9.2) platform for data processing and mass spectral deconvolution. The data processing parameters used in MS-DIAL were as follows: mass accuracy, MS1 and MS2 tolerance were set at 0.02 Da. The *m/z* ranged from 100 to 1500 Da; the minimum peak height was set at 3000 amplitudes, the mass slice width was set at 0.1 Da for peak detection; a sigma window value was set at 0.5; the retention time tolerance was set at 0.025 min, and a QC sample was used as a reference file. For multivariate statistical analysis, the data matrices (peak area) generated from MS-DIAL were imported into Soft Independent Modelling of Class Analogy (SIMCA) software (version 18.0.1) (Sartorius, South Africa). The sample and feature normalisation approaches were applied to reduce redundancy, ensure comparability across variables, and account for measurement error. A seven-fold cross-validation method was applied, and Pareto scaling was used for variable normalisation.

A formal a priori power analysis was not performed. The OPLS-DA model was evaluated using seven-fold cross-validation, explained variance (R^2^), and predictive ability (Q^2^). The statistical significance of the model was assessed using cross-validated analysis of variance (CV-ANOVA), with *p* < 0.05 considered statistically significant. Permutation testing was performed to assess potential model overfitting by comparing the original model with models generated after random permutation of the class labels. Pooled QC sample clustering was used to monitor analytical reproducibility. Missing values were imputed using a non-linear partial least squares algorithm with a correlation factor of 3 and a default threshold of 50%. The PCA and OPLS-DA models were subsequently visualised using score plots. The OPLS-DA loading S-plot was used to identify key discriminant variables based on their covariance and correlation with the model. The selected discriminant variables were further evaluated using variable importance in projection (VIP) scores, with VIP > 1 used as the criterion for confirming variables contributing substantially to group discrimination. Receiver operating characteristic (ROC) analysis was additionally performed within the cross-validation framework to assess classification performance; no independent external validation dataset was used.

### Metabolite annotation and molecular networking

Post MS-DIAL data processing, the Global Natural Products Social Molecular Networking (GNPS) export files, GNPS, MGF files, and feature quantification tables were exported into the GNPS2 ecosystem (https://gnps2.org/homepage) for molecular networking. The networking parameters used for computing Feature-Based Molecular Networking (FBMN) included a precursor ion mass tolerance of 0.02 Da with a fragment ion mass tolerance of 0.02 Da. For spectral similarity, a cosine score cut-off of 0.65 with a minimum of three matched fragment ions was applied. The cosine threshold was selected to retain MS/MS spectral relationships with sufficient fragmentation-pattern similarity for molecular networking while limiting weak or potentially spurious spectral associations. For spectral library annotation, additional stringency was applied by requiring a minimum cosine score of 0.65 and at least six matched peaks. MassBank was used for a spectral library search. To visualise and analyse the networks, the Cytoscape network visualisation software version 3.10.2 (Shannon et al., 2003; Smoot et al., 2011) was used. In addition, manual inspection of the spectral annotations from GNPS2 was done using PubChem (https://pubchem.ncbi.nlm.nih.gov).

The putative annotations were further evaluated by comparison with available literature-reported fragmentation patterns and reported metabolite occurrence. Accordingly, the annotated metabolites were assigned at level 2 of the Metabolomics Standards Initiative (MSI) and are therefore reported as putatively annotated compounds rather than definitive identifications (Sumner et al., 2007). Authentic analytical standards were not analysed in the present study; consequently, MSI level 1 identification cannot be claimed. Future work will prioritise validation of key discriminatory metabolites using authentic standards through comparison of retention times and MS/MS fragmentation patterns acquired under identical analytical conditions.

### Functional analysis and relative quantification

Heatmap and pathway analysis were generated using MetaboAnalyst version 6.0 (https://www.metaboanalyst.ca/). Samples’ peak intensities of all annotated metabolites were imported into statistical analysis (one-factor), a MetaboAnalyst component, and normalised using log transformation and Pareto scaling. For pathway analysis, all annotated metabolites were used for the identification of metabolic pathways and visualisation, and the following parameters were used: (i) visualisation method: scatterplot, (ii) enrichment method: hypergeometric test, (iii) Topology measure: relative betweenness centrality, and (iv) pathway library: *Aspergillus niger* (KEGG). At the time of analysis (December 2024), a *Trichoderma*-specific KEGG pathway library was not available within the MetaboAnalyst pathway-analysis module. Therefore, *A. niger* was selected as an available filamentous ascomycete fungal reference for pathway mapping.

### Compatibility testing between *Trichoderma spp*. and Bacstim®100 in both liquid and solid media

To test the compatibility of *Trichoderma* spp. with Bacstim®100, two culture media were used: half-strength (1) potato dextrose broth (PDB) and (2) tryptic soy broth (TSB), which constituted two sets of experiments. The sterile 250 mL Erlenmeyer flasks were used to prepare the cultures according to Table 1 in triplicate. For TA and TH, the 0.5 g inoculum refers to powdered fungal biomass, whereas for B, the 0.5 g inoculum refers to the commercial *Bacillus* consortium product (Bacstim® 100). The viable microbial concentrations (CFU mL⁻^1^) and fungal spore counts of these inocula were not determined; therefore, the compatibility experiments were standardised on a mass basis rather than on viable cell or spore concentration. The prepared culture flasks were incubated at 30 °C for a total of 7 days. Optical density measurements of TSB flasks were taken at 600 nm to determine the growth rates. After 7 days, the PDB and TSB cultures were centrifuged at 4000 rpm for 40 min, and the supernatants were discarded. The pellets were dried at 60 °C in an incubator and weighed.

**Table 1:** Composition of culture flasks containing 100 mL of PDB or TSB inoculated with TA, TH, and/or B

100 mL of liquid media	TA inoculated (g)	TH inoculated (g)	B inoculated (g)
Control	0	0	0
TA only	0.5	0	0
TA +B	0.5	0	0.5
TH only	0	0.5	0
TH + B	0	0.5	0.5
B only	0	0	0.5

*PDB* potato dextrose broth, *TSB* tryptic soy broth, *TA Trichoderma asperellum* (powdered fungal biomass), *TH Trichoderma harzianum* (powdered fungal biomass), *B*: *Bacillus* consortium (commercial product, Bacstim® 100), *g* grams

Values represent the mass (g) of each product added to the respective treatment flasks

A dual culture plate assay and fermentation broth test were used to investigate the antagonism of the microbial products against each other. To perform a dual culture plate assay, 70 µL of overnight monocultures (prepared according to Table 1) was pipetted into the holes punched on the opposite ends of PDA and TSA plates. For example, a bacterial monoculture was pipetted into the right side of the plate, whereas the fungal product was pipetted into the left side of the plate. The dual plates were then incubated at 30 °C for 7–10 days. Photographs were taken daily to monitor the growth and interaction of the microbes on the plates. For the fermentation broth test, the cultures were prepared according to Table 1, fungal products were prepared in PDB, and bacterial products were prepared in TSB. The flasks were incubated at 30 °C for 7 days. Spent broth was sampled after 24 h and 7 days of incubation. The 10 mL of sampled cultures was centrifuged at 4000 rpm for 30 min, and the supernatants were filtered through a 0.45 µm syringe filter to remove the microbial cells. To investigate the effect of fungal broths on bacterial growth, 1 mL of filtered (24 h and 7 day) spent fungal broths, TA and TH, respectively, was mixed with 25 mL of TSA cooled to 45 °C. To investigate the effect of bacterial broths on fungal growth, 1 mL of filtered (24 h and 7 day) spent bacterial broth was mixed with 25 mL of PDA cooled to 45 °C. After the plates had solidified, 50 µL of TA, TH, and B suspensions, respectively, were pipetted into a hole punched in the middle of the corresponding plates (i.e., TA and TH on PDA plates and B on TSA plates). Unamended PDA and TSA plates were used as controls.

## Results and discussion

Despite increasing interest in microbial compatibility, the metabolic mechanisms that drive synergistic, neutral, or antagonistic interactions among microorganisms remain largely unexplored. This knowledge gap limits the ability to predict and optimize compatible microbial associations. Therefore, there is an urgent need for comprehensive profiling of the metabolome of microorganisms, elucidating the biochemical basis of microbial compatibility, and identifying key metabolic pathways involved in interspecific interactions. The results reported herein are metabolic blueprints of TA and TH. The functional analysis was performed to identify the key metabolic processes and biochemical pathways. Furthermore, in vitro assays were conducted to explore the compatibility between TA, TH, and B.

### Global metabolic blueprints of *T. asperellum* and *T. harzianum*

**Fig. 1:**
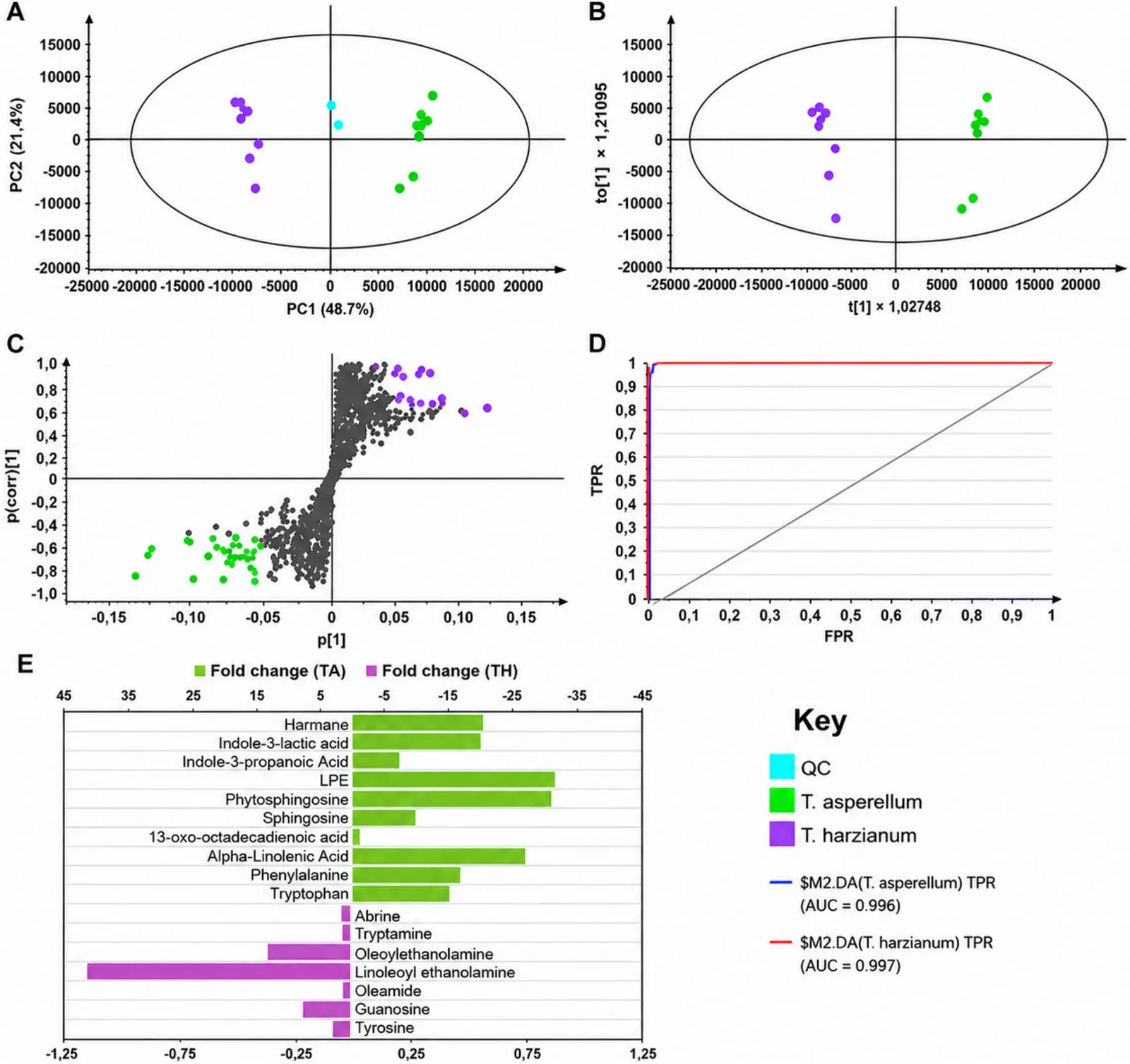
The multivariate statistical methods. Unsupervised Principal Component Analysis (PCA) scores plot revealing the clustering and separation between and within four groups (TA, TH, QC, and control samples), with PC1 and PC2 explaining 72.1% of the variation, R2X (cum) = 0.843, Q2X (cum) = 0.675, and Hotelling’s T2 at 95% confidence interval (**A**). The supervised Orthogonal Partial Least Squares Discriminant Analysis (OPLS-DA) indicates the separation of two groups (TA and TH), R2X (cum) = 0.809, Q2X (cum) = 0.965, p-value < 0.05, at 95% confidence interval (**B**). An OPLS-DA loadings S-plot depicting the key discriminant variables contributing to group separation in the OPLS-DA model, with p [1] ≥ 0.05 and p(corr) ≥ 0.05. (**C**), a receiver operator characteristic (ROC) plot of the OPLS-DA model, showing a summary of the performance of a binary classifier with area under the curve = 1 (**D**), the bidirectional fold change plot highlights metabolites that differed significantly between TA and TH (**E**).* TATrichoderma asperellum*,* THTrichoderma Harzianum*,* QC* quality control

Untargeted metabolomics revealed distinct separation between TA and TH, indicating differential metabolic profiles. The computed principal component analysis (PCA) model displayed robust clustering with clear separation between TA and TH. The pooled QC samples showed close clustering in the PCA score plot (Fig. 1A), supporting stable analytical performance and reproducibility across the LC–MS/MS sequence. Furthermore, PCA comprises cumulative R^2^ (explained variation) of 0.843 and a cumulative Q^2^ (predictive variance) of 0.675, which are above the acceptable values for biological data, i.e., R^2^ > 0.7 and Q^2^ > 0.4 (Godzien et al., 2013). The orthogonal partial least squares discriminant analysis (OPLS-DA) further demonstrated clear separation between TA and TH (Fig. 1B), with a cumulative R^2^ of 0.843 and a cumulative Q^2^ of 0.675. The statistical significance of the OPLS-DA model was supported by CV-ANOVA (*p* < 0.0001). Furthermore, permutation testing showed that none of the permuted models performed better than the original model in separating TA and TH, and no evidence of substantial model overfitting. Together with the seven-fold cross-validation, the R^2^ and Q^2^ values, CV-ANOVA, and permutation testing supported the statistical reliability and predictive performance of the supervised model.

The OPLS-DA loading S-plot was subsequently generated to identify the variables contributing most strongly to the discrimination between TA and TH (Fig. 1C). The cut-off values for the selected key discriminant variables had a covariance, |p[1]| ≥ 0.05, and correlation, |p(corr)| ≥ 0.05, to ensure the selection of variables with combined high covariation and correlation, that are statistically relevant as potential discriminant features (Tugizimana et al., 2013; Wiklund et al., 2008). Receiver operating characteristic (ROC) analysis was performed using the cross-validation dataset to assess classification performance (Fig. 1D). The ROC analysis yielded an AUC of 1.00, indicating strong discriminatory performance between TA and TH within the cross-validation framework. However, because an independent external validation dataset was not used, this value should be interpreted as an internal estimate of classification performance rather than evidence of external predictive performance (Hamany Djande et al., 2021).

Following LC–MS data processing, a total of 3,215 features were detected across the analysed samples. Of these, 42 features were putatively annotated at MSI level 2, representing approximately 1.31% of the detected feature space (Supplementary Table S1). The remaining 3173 features (98.69%) remained unannotated under the spectral matching and annotation criteria applied in this study. Of the 42 putatively annotated metabolites, 17 were subsequently selected as key discriminants between TA and TH (Supplementary Table S2; Fig. 1E) (Supplementary Table 2, Fig. 1E). The direction and relative magnitude of differences in these selected metabolites between TA and TH were subsequently visualised using a bidirectional fold-change plot (Fig. 1E); fold change was not used as an independent selection threshold. These metabolites largely belonged to three functional classes: aromatic amino acids, indole derivatives, and lipid metabolites, underscoring differences in primary metabolism, signalling, and membrane composition. TA was characterised by elevated levels of tryptophan, phenylalanine, indole-3-lactic acid, indole-3-propanoic acid, sphingosine, and phytosphingosine. In contrast, TH accumulated higher levels of tyrosine, guanosine, tryptamine, and linoleoyl ethanolamide (Supplementary Table 2; Fig. 1E). This differential distribution highlights distinct metabolic strategies, with TA enriched in lipid-associated metabolites and TH biased towards indole and nitrogen-related compounds.

#### Differential metabolic profiles between *T. asperellum* and *T. harzianum*

**Fig. 2:**
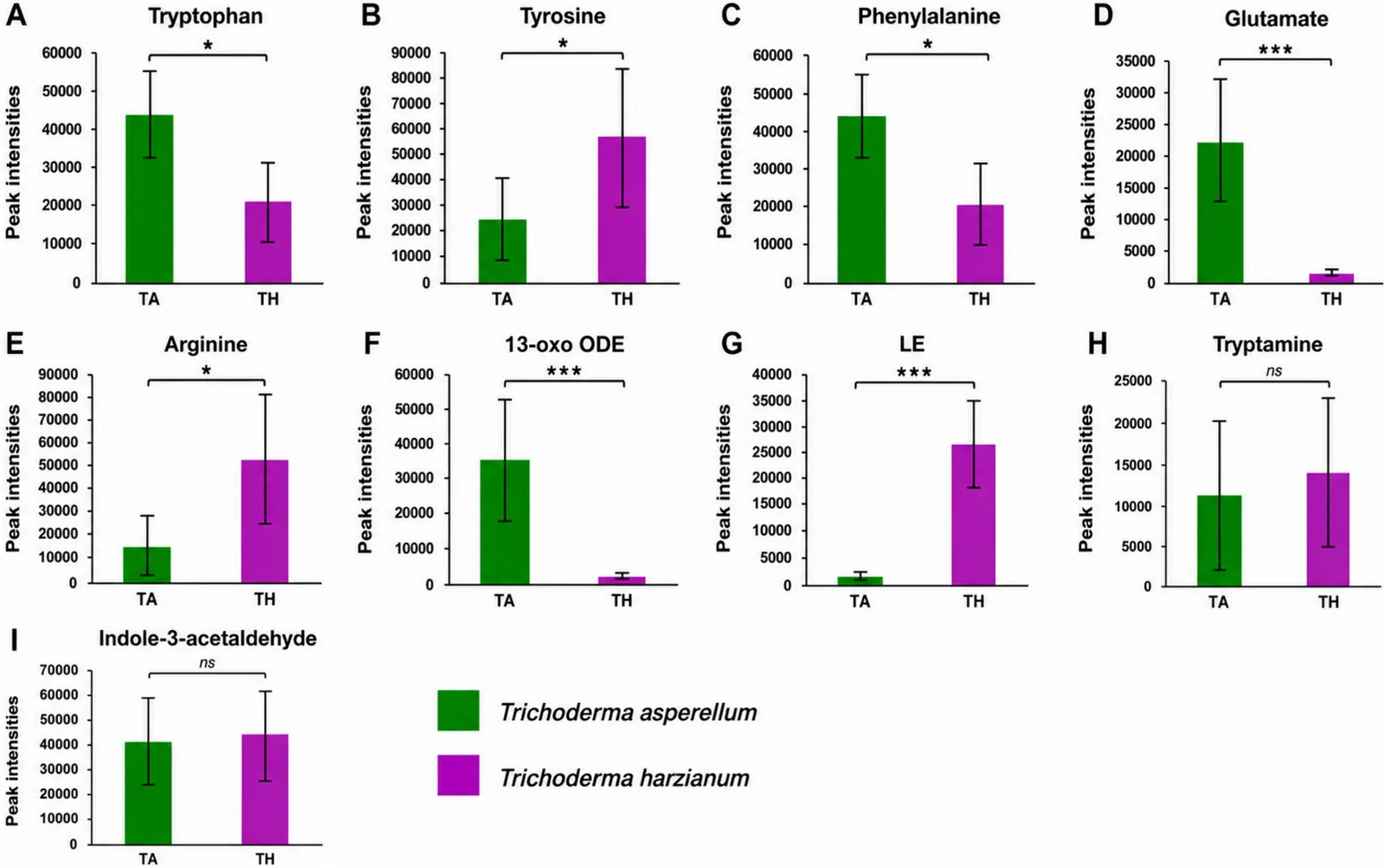
Relative quantification of amino acids. The bar graphs showing the quantification levels of tryptophan (**A**), tyrosine (**B**), phenylalanine (**C**), glutamate (**D**), arginine (**E**), 13-oxo ODE (**F**), LE (**G**), tryptamine (**H**), and indole−3-acetaldehyde (**I**) were generated using the averaged peak intensities (n = 9), and the error bars represent the standard deviation. The differences in statistical significance of metabolites between TA and TH are denoted by asterisks (*), where *p-*values ≤ 0.05 and *p-values* ≤ 0.001 are denoted by * and ***, respectively. *TA Trichoderma asperellum*,* THTrichoderma harzianum,* and* ns* non-significant

The results revealed clear metabolomic differences between TA and TH, indicating that each species possesses a distinct metabolomic signature despite belonging to the same genus. The observed differences in metabolomic signatures are predominantly associated with alterations in amino acids, indoles, and lipids. The amino acids, including tryptophan, tyrosine, phenylalanine, glutamate, and arginine, were differently regulated between TA and TH (Fig. 2). TA exhibited higher levels of tryptophan and phenylalanine, whereas TH exhibited higher levels of tyrosine. Tryptophan, tyrosine, and phenylalanine serve as biological precursors for a broad range of secondary metabolites, involved in signaling and environmental adaptation (Réthoré et al., 2021). The higher accumulation of tryptophan in TA and the increased abundance of tryptamine and indole-3-acetaldehyde in TH suggest differential utilization of tryptophan metabolism between TA and TH. As tryptophan serves as a precursor for biosynthesis of indoles, its lower levels in TA may reflect conversion, whereas the enrichment of tryptamine and indole-3-acetaldehyde (IAL) in TH indicates enhanced downstream metabolism. The IAL can be oxidised to indole-3-acetic acid (IAA) using alcohol dehydrogenase enzyme, making IAL a precursor to IAA, which is known for its ability to start the microbial auxin biosynthesis pathway (Lee et al., 2025; Tang et al., 2023; Vega-Celedón et al., 2024). Furthermore, IAA promotes fungal growth, induces spore germination, and elongates fungal hyphae (Choub et al., 2025). A recent study highlighted that *Trichoderma* sp.*18–13* is effective in producing IAA (Karaca & Eltem, 2026).

The glutamate levels were higher in TA, whereas arginine levels were higher in TH (Fig. 2). Glutamate can be modified into glutamine, which is utilised by fungi as a source of nitrogen (Pang et al., 2021). In contrast, arginine serves primarily as a key nitrogen storage and transport molecule, allowing fungi to regulate intracellular nitrogen levels and redistribute nitrogen according to metabolic demands (Yang et al., 2024). A study where *Aspergillus niger* was cultured in carboxymethylcellulose (CMC) revealed the accumulation of arginine and highlighted that arginine plays a key role in conidiation and supports *Aspergillus niger* to adapt to CMC (Borin and Oliveira, 2022). Thus, the elevated arginine levels observed in TH may reflect altered nitrogen metabolism. Lipid-derived metabolites also contributed to strain differentiation. TH exhibited higher levels of linoleoyl ethanolamide (LE), whereas TA exhibited higher levels of 13-Oxo-ODE (Fig. 2). LE and 13-oxoODE are classified as polyunsaturated fatty acids, playing a crucial role in cells by altering cell membrane integrity (Carbonell-Rozas et al., 2025). These lipid-associated metabolites may contribute to membrane-associated stress responses and cellular homeostasis in *Trichoderma* spp. Thus, these findings demonstrate distinct chemical profiles associated with the TA and TH materials under the experimental conditions investigated.

Furthermore, although methanol enabled the detection of diverse metabolite classes in the present study, including lipid-associated compounds, the use of complementary non-polar or biphasic extraction systems could provide broader coverage of highly hydrophobic metabolites and may be considered in future studies aimed at comprehensive lipidomic characterisation. In addition, another important consideration in interpreting these findings is that metabolomic profiling was performed on powdered fungal biomass rather than freshly harvested fungal cultures. Although the analysed materials did not contain formulation carriers or excipients, biomass processing and storage may influence the stability and relative abundance of some metabolites through processes such as degradation or oxidation. Therefore, the metabolomic profiles reported here represent the chemical composition of the powdered *Trichoderma* biomass under the conditions investigated. Future studies comparing freshly harvested and stored fungal biomass would be valuable for assessing the stability of individual metabolic markers during processing and storage.

#### Functional analysis: differential biological pathways in *T. asperellum* and *T. harzianum*

**Fig. 3:**
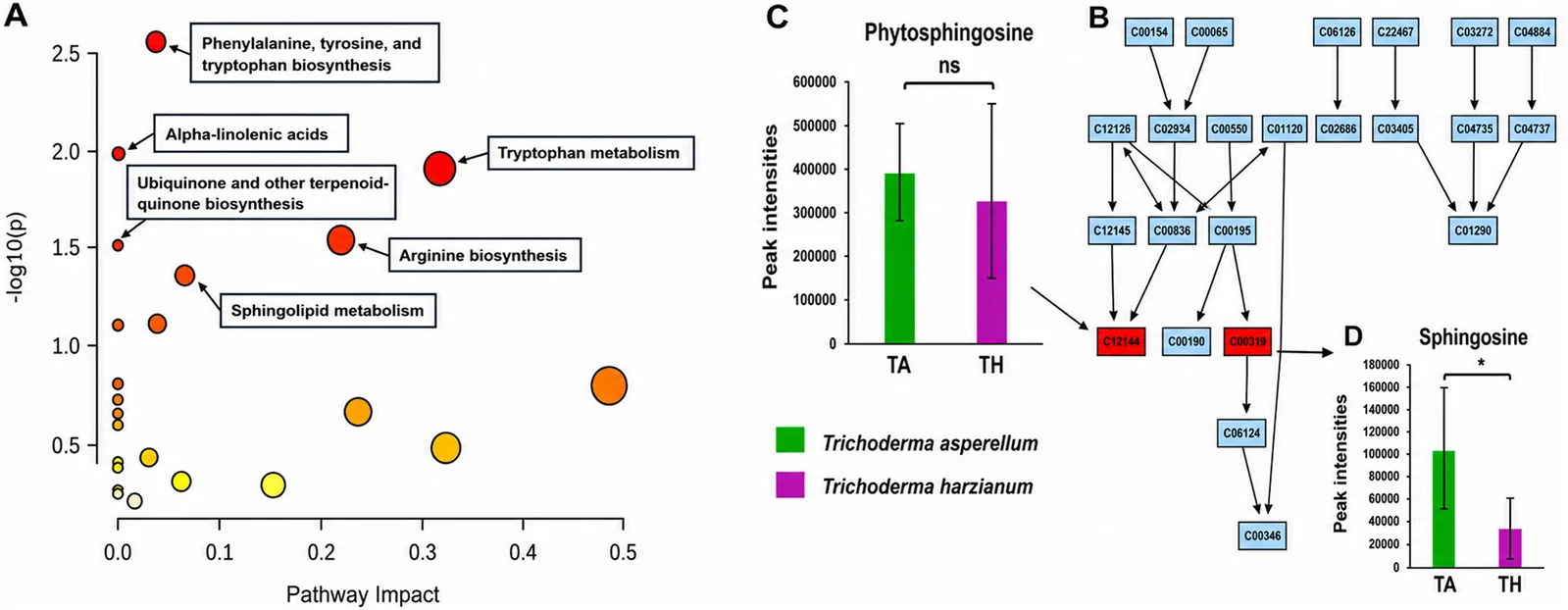
Metabolic pathway and relative quantification. The pathway analysis shows all matched, impacted pathways (**A**), ranked by pathway impact on the y-axis and p-values on the x-axis. Each node corresponds to the mapped metabolic pathways. The node with red colour indicates the highest significance, whereas the nodes with yellow colour indicate the lowest significance. The node size indicates pathway impact: larger nodes represent greater influence. The -log(p) and impact of all labelled impacted pathways are shown in Table S3. A topological presentation of sphingolipid metabolism extracted from pathway analysis (**B**). The bar graphs showing the quantification levels of phyto-sphingosine (**C**), and sphingosine (**D**) were generated from averaged peak intensities (n = 9), and the error bars represent the standard deviation. The differences in statistical significance of metabolites between TA and TH are denoted by asterisks (*), where *p*-value ≤ 0.05 and 0.001 are denoted by * and ***, respectively; ns=non-significant. Heatmaps showing the levels of all annotated lipids (**F**). The full names of metabolites are in Table S1.* TATrichoderma asperellum,* and* THTrichoderma harzianum*

To explore the metabolic pathways potentially associated with the observed differences between the two *Trichoderma* strains, all putatively annotated metabolites (Supplementary Table S1) were subjected to pathway analysis. Metabolic pathway analysis provides a clearer understanding of how these molecular changes affect important cellular processes, such as energy metabolism and secondary metabolite biosynthesis (Rinschen et al., 2019). Furthermore, this comprehensive viewpoint offers important insight into the functional effects of molecular changes. The metabolic pathways were plotted according to their statistical significance (− log(*p*-value) on the *y*-axis and their corresponding pathway impact scores on the* x*-axis, reflecting enrichment and topology analyses, respectively (Fig. 3A, Supplementary Table S3). Pathway analysis indicated several significantly enriched metabolic pathways, including sphingolipid metabolism, purine metabolism, phenylalanine metabolism, phenylalanine, tyrosine and tryptophan biosynthesis, ubiquinone and other terpenoid-quinone biosynthesis, tyrosine metabolism, and tryptophan metabolism. Sphingolipid metabolism (Fig. 3B) revealed a pronounced impact, driven by elevated levels of phytosphingosine (Fig. 3C) and sphingosine (Fig. 3D) in TA. Sphingolipids play a key role in several biological processes of a fungus, including cell division, hyphal formation, and spore germination for their survival in any given environmental conditions (Levery et al., 2002; Oura & Kajiwara, 2010; O‘Connell et al., 2012). Phytosphingosine contributes to membrane stability and cellular functions, whereas sphingosine acts as a signalling molecule involved in regulating cellular processes such as cell differentiation and proliferation (Fernandes et al., 2018; Kitamura et al., 2017). The elevated levels of phytosphingosine and sphingosine in TA could be associated with enhanced membrane stability and adaptive capacity, which may confer advantages under fluctuating environmental conditions.

### Evaluating the compatibility between *Trichoderma* strains and the *Bacillus* consortium

**Fig. 4:**
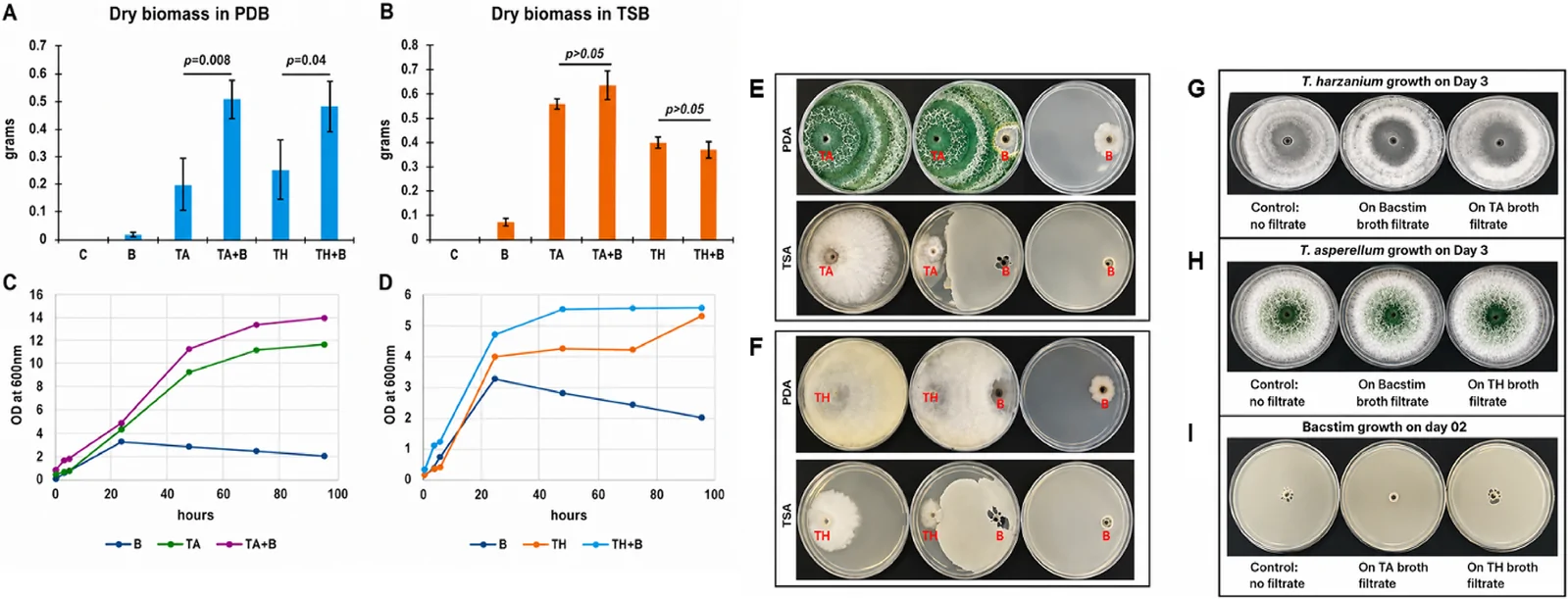
Biomass and optical density curves of *Trichoderma* spp. and *Bacillus* consortium. Bar graphs showing dry biomass of microbial products grown in PDB and TSB as monoculture vs. co-cultures (**A**, **B**). Growth curves of the microbial products grown in TSB as monoculture vs. co-cultures (**C**, **D**). Dual plate assay showing the interaction between *T. asperellum* and *Bacillus* consortium (**E)**; and *T. harzianum* and *Bacillus* consortium (**F**) on PDA and TSA. Fermentation broth test − 24 h spent broth filtrate of *T. harzianum* on *Bacillus* consortium and *T. asperellum* (**G**), *T. asperellum* on *T. harzianum* and *Bacillus* consortium (**H**), Bacillus consortium on *T. asperellum* and *T. harzianum*. Control plates with no filtrate were also included. *TAT.asperellum*,* THT. harzianum*,* B* Bacstim®100,* PDB* potato dextrose broth,* TSB* tryptic soy broth,* OD* optical density

The compatibility evaluation of microorganisms is a key requirement for the development of stable and effective microbial consortia biostimulants. In the present study, the observed interaction patterns revealed varying degrees of compatibility among TA, TH, and B, suggesting that microbial coexistence is governed by strain-specific physiological traits. Co-culture experiments demonstrated that TA and TH are compatible with B. In PDB medium, co-cultivation significantly increased biomass in both TA (+ 168.4%) and TH (+ 98.6%) compared to monocultures (Fig. 4A). In TSB medium, growth differences were minor and not statistically significant (Fig. 4B). However, growth curves indicated comparable, improved proliferation in co-cultures (Fig. 4C, D). Although total dry biomass was higher in the PDB co-cultures than in the corresponding monocultures, this increase cannot be attributed to synergistic growth because the recovered biomass represents the combined biomass of both microorganisms. The present experimental design did not quantify the individual contribution of each microbial partner or test whether co-culture growth exceeded an expected additive response. In addition, the inocula were standardised by mass rather than by viable cell or spore concentration. Consequently, potential differences in viable microbial density between the fungal biomass and commercial bacterial preparation could not be accounted for and should be considered when interpreting the magnitude of the observed growth responses. The increased biomass is therefore interpreted as an additive growth effect consistent with microbial compatibility rather than evidence of true synergy.

Furthermore, the dual culture assays confirmed minimal antagonism (Fig. 4E, F). A transient inhibition zone was observed for TA at day 3, yet disappeared by day 10, indicating recovery and coexistence (Supplementary Fig S1). No inhibition was observed between TA and B (Fig. 4E). The interaction between TH and B (Fig. 4F) did not show any inhibition band on either TSA or PDA. The fermentation broth assay demonstrated an absence of antagonistic interactions among either TA or TH with B (Fig. 4G–I and Supplementary Fig S2). The co-cultivation of TH with B and TA (Fig. 4G), TA with B and TH (Fig. 4H), and B with TA and TH (Fig. 4I) revealed no evidence of growth inhibition, reduced viability, or suppression of metabolic activity, indicating that TA, TH, and B maintained stable growth dynamics under shared culture conditions. The lack of inhibitory interactions supports the ability of the microorganisms to coexist under the tested culture conditions. However, because the assays were designed primarily to assess compatibility and antagonism, these observations should not be interpreted as evidence of microbial synergy. Such compatibility is critical for the development of stable and effective microbial consortia, as antagonistic interactions can compromise formulation stability and reduce field performance (Samonty et al., 2025; Wu et al., 2023). Previous studies have shown that *Trichoderma* and *Bacillus* occupy complementary niches within the rhizosphere and can synergistically enhance plant growth, nutrient uptake, and disease suppression (Uribe-Acosta et al., 2025; Xie et al., 2025). The findings presented here are consistent with these observations and further provide a metabolomic context to support functional compatibility.

**Fig. 5:**
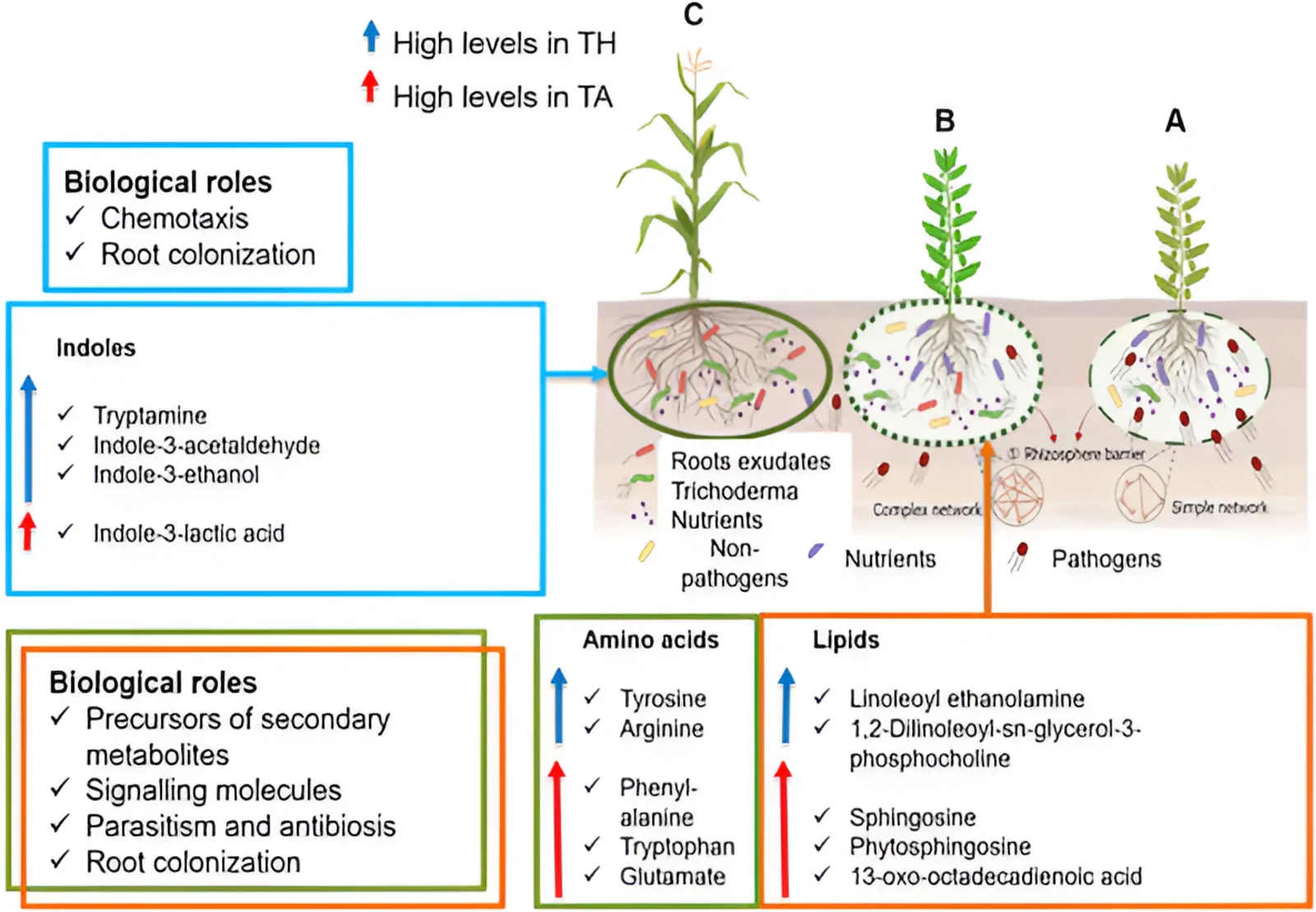
Conceptual representation of the potential biological relevance of the metabolic profiles of *Trichoderma* spp. The metabolomic profiles of *T. asperellum* and *T. harzianum* revealed several chemical classes, including amino acids, lipids, and indole-related metabolites. Based on biological functions previously reported in the literature, these metabolite classes may be associated with fungal growth and development and may have potential relevance to plant–microbe interactions. The figure illustrates the native rhizosphere environment in the absence of *Trichoderma* application (**A**) and the potential influence of *Trichoderma* on rhizosphere microbial and plant–microbe interactions following application (**B**). Reported interactions of *Trichoderma* within the rhizosphere include competition for nutrients, modulation of root-associated processes, and production of bioactive compounds that may influence microbial community composition and activity (**C**). In the present study, TA exhibited comparatively higher levels of several lipid-associated metabolites, whereas TH showed higher levels of some indole-related metabolites. These species-associated metabolic differences may indicate distinct functional potential relevant to environmental adaptation and plant–microbe interactions. However, the proposed plant-related functions illustrated here are literature-supported hypotheses and were not directly evaluated in plants in the present study.* BBacillus* consortium;* TATrichoderma asperellum*;* THTrichoderma harzianum*

To contextually summarize the findings, the metabolite profiles observed in this study have important implications for plant–microbe interactions (Fig. 5). Amino acids such as arginine have previously been associated with nitrogen metabolism and signalling processes relevant to rhizosphere microbial activity and plant responses (Pang et al., 2021; Yang et al., 2024). Similarly, indole derivatives and intermediates associated with auxin biosynthesis have been reported to influence plant growth and development. Previous studies have linked microbial production of indole-3-acetaldehyde and related indole compounds with processes such as root development, nutrient acquisition, and plant defence responses (Vega-Celedón et al., 2024; Zhang et al., 2025). Therefore, the comparatively higher abundance of some indole-related metabolites in TH may indicate potential functional relevance to plant–microbe interactions. Likewise, the lipid- and sphingolipid-associated profile observed in TA may be relevant to fungal membrane stability, environmental adaptation, and potentially root-associated persistence (Carbonell-Rozas et al., 2025). However, these proposed plant-related functions remain hypotheses based on previously reported biological roles of these metabolite classes, as plant responses were not experimentally evaluated in the present study.

Furthermore, previous studies have reported that the combined use of *Trichoderma* and *Bacillus* can produce synergistic effects that surpass the benefits of individual inoculation (Kumari et al., 2025). However, the present study demonstrates compatibility rather than synergy, as the experimental design did not quantify whether the combined response exceeded an expected additive effect. This synergy arises from their ability to colonise different ecological niches in the rhizosphere: *Trichoderma* predominates at the soil-root interface, and *Bacillus* thrives in the rhizoplane and surrounding soil (Shahriar et al., 2022; Uribe-Acosta et al., 2025). In combination, *Trichoderma* and *Bacillus* create a more functionally diverse and resilient microbiome, capable of stimulating root development, providing enhanced protection against soil-borne pathogens, and improving nutrient uptake (Ansabayeva et al., 2025). These traits are essential for sustained microbial establishment and biocontrol efficacy. Thus, the observed metabolic divergence suggests complementary functional roles, supporting the rationale for combining these strains in multi-component biostimulant formulations.

## Conclusion and perspectives

This study expands the chemical knowledge of *Trichoderma asperellum* and *T. harzianum* through comparative untargeted LC–MS/MS metabolomics, multivariate analysis, molecular networking, and pathway analysis. The two species displayed distinct metabolomic signatures, with key differences associated predominantly with amino acid, indole, and lipid metabolism. These findings provide a biochemical basis for distinguishing the two *Trichoderma* materials and add information on species-associated chemical characteristics and metabolic processes relevant to fungal physiology and environmental adaptation. The identified metabolites also establish a reference point for future investigations of *Trichoderma* chemical composition. Although several of these metabolite classes have previously been associated with plant–microbe interactions, their proposed roles in plant growth promotion, nutrient acquisition, and plant defence remain hypotheses because plant responses were not directly evaluated in this study. Importantly, the chemical characterisation was complemented by experimental assessment of compatibility with a defined *Bacillus* consortium. Both *T. asperellum* and *T. harzianum* coexisted with the *Bacillus* consortium under the tested culture conditions without substantial antagonistic interactions. Increased total biomass observed in some co-cultures was consistent with compatibility and additive growth rather than demonstrated microbial synergy, as the individual contributions of the fungal and bacterial partners were not resolved. The integration of metabolomic characterisation with compatibility assessment therefore provides information beyond species differentiation alone by linking the chemical characteristics of the *Trichoderma* materials with their suitability for further evaluation in multi-microbial systems.

From a biostimulant-development perspective, this chemical information can contribute to a growing knowledge base for microbial material characterisation, strain and consortium selection, product development, and future quality assessment. The discriminant metabolites identified here provide candidate chemical markers that can be evaluated for distinguishing *Trichoderma*-based materials and monitoring compositional consistency. Such chemical markers may be particularly relevant to an industry in which variation in microbial composition, production processes, and formulation can affect product consistency and performance. Their practical application, however, will require validation across additional strains, production batches, formulations, and independent sample sets before they can be applied to unknown or complex biostimulant materials.

Future research should integrate metabolomic profiling with controlled plant experiments to establish whether the observed chemical differences translate into differential biostimulant effects *in planta*. Metabolomic investigation of *Trichoderma–Bacillus* co-cultures could further reveal chemical interactions that are not apparent from individual microorganisms. Compatibility studies should also standardise inocula using viable cell and spore concentrations and employ species-specific quantitative approaches to distinguish additive, synergistic, and antagonistic responses. These advances can support more informed microbial selection, formulation design, compositional standardisation, and evidence-based development of multi-microbial biostimulants.

## Supplementary Information

Below is the link to the electronic supplementary material.

Supplementary Material 1

## Data Availability

The GNPS molecular networking job links associated with this study are provided in the Supplementary Information. The raw LC–MS data are available from the corresponding author upon request. Deposition of the raw LC–MS dataset in a public repository is currently in progress.
