# Supplementary material for: The chemical blueprint of Trichoderma for next-generation microbial biostimulants
Source: Metabolomics. 2026 Oct 3;22(5):166. doi: 10.1007/s11306-026-02531-4 (PMC13633993; doi:10.1007/s11306-026-02531-4)
Supplement: Supplementary file 1 [file 11306_2026_2531_MOESM1_ESM.pdf]

*Supplementary material*

# The Chemical Blueprint of *Trichoderma* for Next-Generation Microbial Biostimulants

Thuso Mudau[1], Lerato Pertunia Tshehlane[1], Keabetswe Tebogo Ncube[1], Fidele Tugizimana[1,2]*

**GNPS FBMN link:**

https://gnps.ucsd.edu/ProteoSAFe/status.jsp?task=8e8ee443c8d74b6c8b6dc26d67398d53

**GNPS2 FBMN Link:**

https://gnps2.org/result?task=a81ac9db8b8e4bf0ab87e1bb4915e106&viewname=librarymatches&resultdisplay_type=task

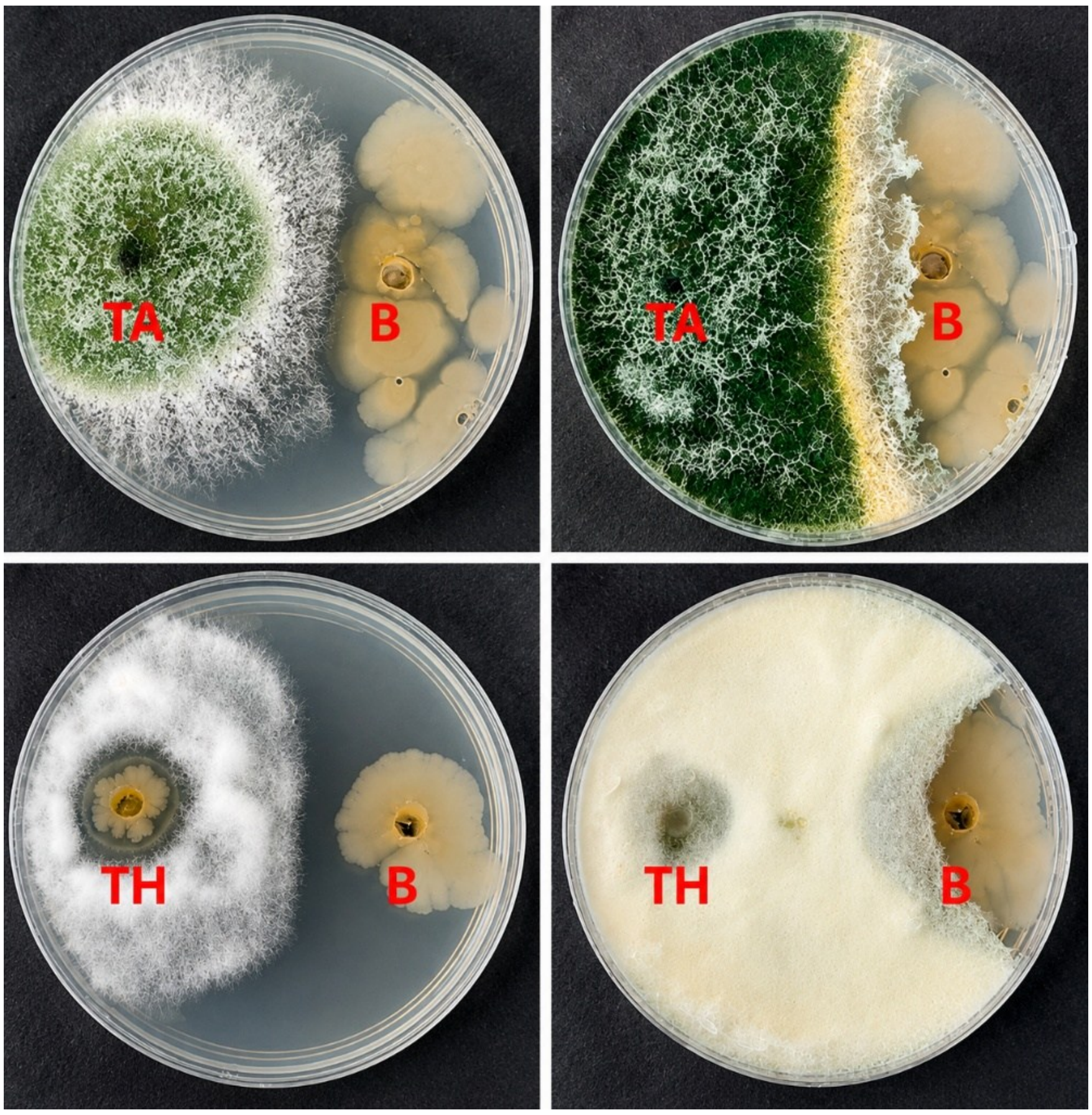


**Figure S1:** Dual plate assay showing the interaction between *Trichoderma spp*. and *Bacillus* consortium on PDA and TSA. Abbreviations: TA = *T.asperellum*, TH = *T. harzianum*, and B = *Bacillus* consortium.

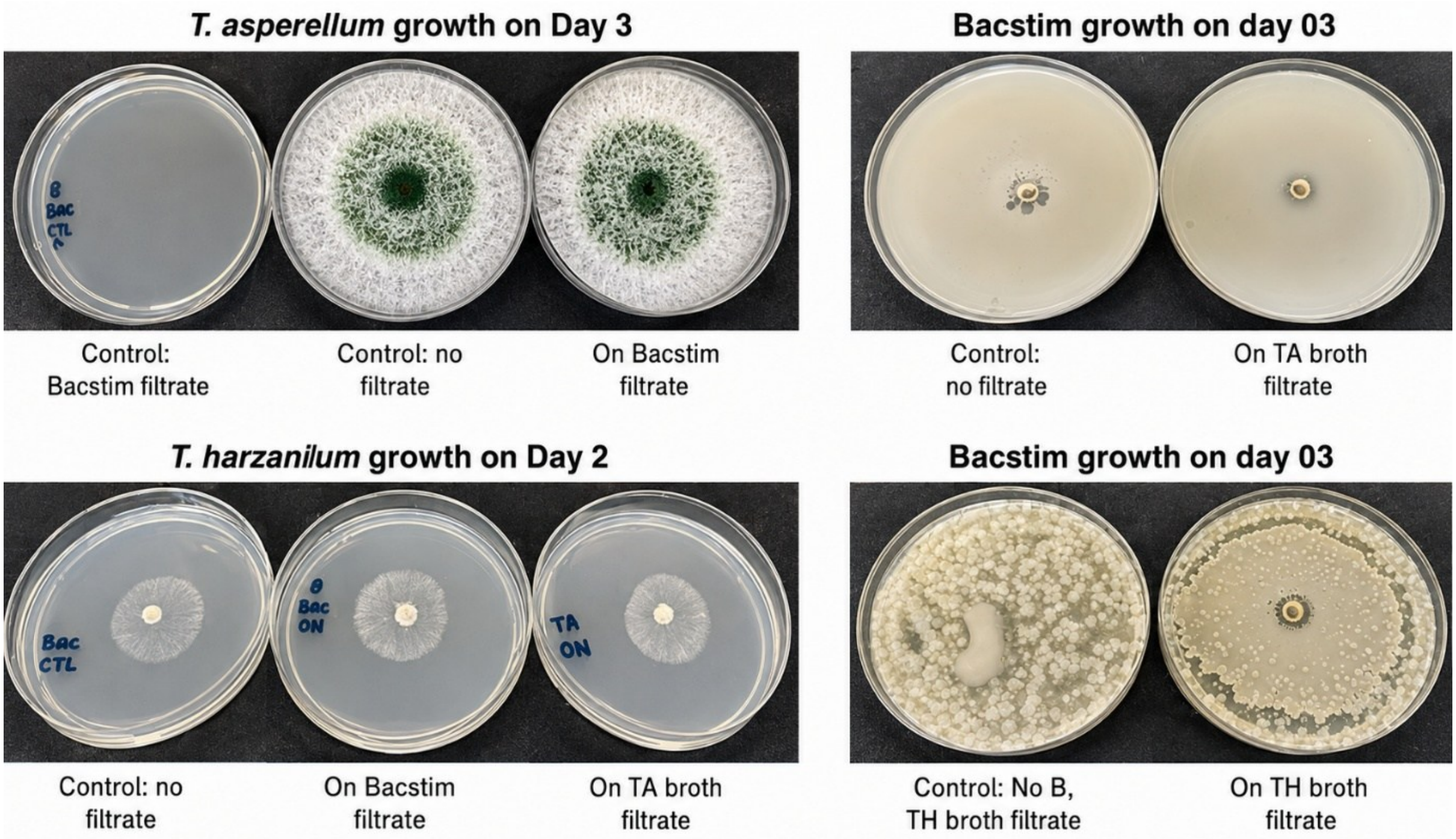


**Figure S2:** Fermentation broth test. Evaluation of the culture filtrate harvested after 7 days of fermentation. Abbreviations: TA = *T.asperellum*, TH = *T. harzianum*, and B = *Bacillus* consortium.

**Table S1:** List of putatively annotated metabolites from *T. asperellum* and *T. harzianum*.

| Compound name | Abbreviation | Chemical formula | m/z | Rt (min) | Fragments | Adduct |
|---|---|---|---|---|---|---|
| **Phenylalanine** | Phe | $C_9H_{11}NO_2$ | 166.08 | 3.483 | 103; 120; 121 | [M+H]+ |
| **Tyrosine** | Tyr | $C_9H_{11}NO_3$ | 182.08 | 1.645 | 119; 123; 136 | [M+H]+ |
| **Tryptophan** | Trp | $C_{11}H_{12}N_2O_2$ | 205.09 | 4.04 | 118; 144; 146; 188 | [M+H]+ |
| **Glutamate** | Glu | $C_5H_9NO_4$ | 148.06 | 8.608 | 102; 130 | $[[M+H]+]^+$ |
| **Arginine** | Arg | $C_6H_{14}N_4O_2$ | 175.11 | 2.992 | 116; 130; 158 | [[M+H]+]+ |
| **N-Acetylleucine** | Ace-Leu | $C_8H_{15}NO_3$ | 174.11 | 9.51 | 128 ; 132 ; 156 | [[M+H]+]+ |
| **Threonylleucine** | Thre-Leu | $C_{10}H_{20}N_2O_4$ | 233.15 | 12.304 | 132; 187; 215 | [[M+H]+]+ |
| **Cyclo(Leu-Pro)** | Cyclo (Leu-pro) | $C_{11}H_{18}N_2O_2$ | 211.14 | 5.965 | 138 ; 155 ; 183 ; 194 | [[M+H]+]+ |
| **ValylIsoleucine** | Val-Leu | $C_{11}H_{22}N_2O_3$ | 231.17 | 6.072 | 120 ; 132 ; 205 | [[M+H]+]+ |
| **Methylthioadenosine** | Meth-Ade | $C_{11}H_{15}N_5O_3S$ | 298.09 | 4.144 | 61 ; 75; 97; 136; 145 | [M+H]+ |
| **THYMINE** | Thymine | $C_5H_6N_2O_2$ | 127.05 | 4.037 | 110; 109; 84; 56 | [M+H]+ |
| **Guanosine** | Guo | $C_{10}H_{13}N_5O_5$ | 284.1 | 3.011 | 153; 152; 135; 110 | [M+H]+ |
| **Alpha-linolenic acid** | ALA | $C_{18}H_{30}O_2$ | 279.23 | 12.65 | 81; 95; 243; 261; 279 | [M+H]+ |
| **13-oxo-octadecadienoic acid** | 13-HODE | $C_{18}H_30O_3$ | 295.23 | 9.67 | 179; 259; 277 | [[M+H]+]+ |

| | | | | | | |
|---|---|---|---|---|---|---|
| **Oleamide** | Oleamide | $C_{18}H_{35}NO$ | 282.28 | 15.292 | 135 ; 247 ; 265 | [M+H]+ |
| **Linoleoyl ethanolamide** | LE | $C_{20}H_{37}NO_2$ | 324.29 | 14.049 | 263; 306; 307; 324 | [M+H]+ |
| **Oleoyl ethanolamine** | OE | $C_{20}H_{39}NO_2$ | 326.31 | 12.826 | 308; 309; 328; 326 | [M+H]+ |
| **Sphingosine** | Sph | $C_{18}H_{37}NO_2$ | 300.29 | 9.985 | 282; 283; 252; 211 | [M+H]+ |
| **Phytosphingosine** | Phytosph | $C_{18}H_{39}NO_3$ | 318.30 | 9.997 | 282; 300; 318 | [M+H]+ |
| **1,2-Dilinoleoyl-sn-glycero-3-phosphocholine** | DLPC | $C_{44}H_{80}NO_8P$ | 782.58 | 14.263 | 184; 185; 124; 425 | [M+H]+ |
| **1-palmitoyl-2-hydroxy-sn-glycero-3-phosphoethanolamine** | LPE | $C_{24}H_{44}NO_7P$ | 454.30 | 12.704 | 478; 184; 104 | [M+H]+ |
| **5-hydroxyindole** | Hyd-ind | $C_8H_7NO$ | 134.06 | 3.265 | 106 ; 79; 71 | [M+H]+ |
| **1-methoxyindole-3-carbaldehyde** | MCL | $C_{10}H_9NO_2$ | 176.07 | 8.612 | 116; 133; 148; 161 | [M+H]+ |
| **Indoline** | Indoline | $C_8H_{11}NO$ | 120.08 | 3.475 | 91; 93; 103; 118 | M-H2O+H |
| **Indole-3-propionic acid** | IPA | $C_{11}H_{11}NO_2$ | 190.09 | 7.652 | 130; 131; 172; 173 | {M+H]+ |
| **Indole-3-lactic acid** | ILA | $C_{11}H_{11}NO_3$ | 188.07 | 4.038 | 118; 144; 146 | M+H-H2O |
| **Indole-3-ethanol** | IEt | $C_{10}H_{11}NO$ | 144.08 | 4.443 | 115 ; 117 ; 128 | M+H-H2O |
| **Tryptamine** | Trt | $C_{10}H_{12}N_2$ | 161.11 | 4.37 | 115; 117; 127; 143; 144 | [M+H]+ |
| **Indole-3-acetaldehyde** | IAL | $C_{10}H_9NO$ | 160.08 | 5.218 | 117; 118; 130; 160 | [M+H]+ |

| | | | | | | |
|---|---|---|---|---|---|---|
| **Myosmine** | Myo | $C_9H_{10}N_2$ | 147.09 | 5.808 | 115; 83; 69 | [M+H]+ |
| **Harmane** | HM | $C_{12}H_{10}N_2$ | 183.09 | 5.427 | 115; 142; 168 | [M+H]+ |
| **Abrine** | ABR | $C_{12}H_{14}N_2O_2$ | 219.11 | 5.679 | 118; 132; 144 ; 146; 188 | [M+H]+ |
| **Pilocarpine** | PIL | $C_{11}H_{16}N_2O_2$ | 209.13 | 3.02 | 95; 96; 163; 191 | [M+H]+ |
| **Nornicotine** | NOR | $C_9H_{12}N_2$ | 149.11 | 4.682 | 106; 117; 120 | [M+H]+ |
| **4-phenylpyridine** | 4-PhPy | $C_{11}H_9N$ | 156.08 | 5.391 | 78; 96; 128; 129; | [M+H]+ |
| **1,5-Naphthalenediamine** | NAD | $C_{10}H_{10}N_2$ | 159.09 | 4.039 | 142; 143; 158 | [M+H]+ |
| **Phenyl ethanolamine** | PEOH | $C_8H_{11}NO$ | 120.08 | 3.475 | 91; 93; 103 ; 118 | M-H2O+H |
| **Simetryn** | SIM | $C_8H_{15}N_5S$ | 214.11 | 4.87 | 124 ; 144 ; 166 ; 186 | [M+H]+ |
| **Metribuzin-diketo** | DK | $C_7H_{12}N_4O_2$ | 185.10 | 9.933 | 101; 141; 156; 157; 169 | [M+H]+ |
| **Isovalerylcarnitine** | C5 | $C_{12}H_{23}NO_4$ | 246.17 | 4.682 | 187; 85. ; 60 | [M+H]+ |
| **4,8-dihydroxy-6-methoxy-3-methyl-3,4-dihydroisochromen-1-one** | DHMC | $C_{11}H_{12}O_5$ | 225.08 | 5.48 | 118; 133; 179; 207 | [M+H]+ |
| **6-Quinolinol** | 6HQ | $C_9H_7NO$ | 146.06 | 4.039 | 117; 118; 128 | [M+H]+ |

**Table S2:** A summary of differentially metabolites

| Metabolites | *m/z* | Rt (min) | Adduct | Chemical formula | *p*-value | Fold change | Positively Correlated to |
|---|---|---|---|---|---|---|---|
| **Tyrosine** | 182.083 | 1.64 | M+H | $C_9H_{11}NO_3$ | 0.0065924 | 2.69879 | TH |
| **Guanosine** | 284.101 | 3.003 | M+H | $C_{10}H_{13}N_5O_5$ | 4.0366E-7 | 7.34787 | TH |
| **Oleamide** | 282.285 | 15.292 | M+H | $C_{18}H_{35}NO$ | 0.006373 | 1.13298 | TH |
| **Linoleoyl Ethanolamide** | 324.294 | 14.048 | M+H | $C_{20}H_{37}NO_2$ | 4.3561E-7 | 41.2649 | TH |
| **Oleoylethanolamine** | 326.31 | 12.826 | M+H | $C_{20}H_{39}NO_2$ | 0.0071343 | 12.9678 | TH |
| **Tryptamine** | 161.11 | 4.37 | M+H | $C_{10}H_{12}N_2$ | 0.0051012 | 1.2937 | TH |
| **Abrine** | 219.106 | 5.679 | M+H | $C_{12}H_{14}N_2O_2$ | 1.3061E-4 | 1.46594 | TH |
| **Tryptophan** | 205.099 | 4.04 | M+H | $C_{11}H_{12}N2O_2$ | 0.0064875 | 0.422403 | TA |
| **Phenylalanine** | 166.089 | 3.483 | M+H | $C_9H_{11}NO_2$ | 6.7685E-4 | 0.471022 | TA |
| **Alpha-Linolenic Acid** | 279.233 | 12.65 | M+H | $C_{18}H_{30}O_2$ | 0.021814 | 0.750668 | TA |
| **13-oxo-octadecadienoic acid** | 295.23 | 9.672 | M+H | $C_{18}H_{30}O_3$ | 8.8979E-6 | 0.0217179 | TA |
| **Sphingosine** | 300.295 | 9.899 | M+H | $C_{18}H_{37}NO_2$ | 0.010625 | 0.275379 | TA |
| **Phytosphingosine** | 318.304 | 9.985 | M+H | $C_{18}H_{39}NO_3$ | 0.045972 | 0.858427 | TA |
| **1-palmitoyl-2-hydroxy-sn-glycero-3-phosphoethanolamine** | 454.302 | 12.704 | M+H | $C_{21}H_{44}NO_7P$ | 0.0043222 | 0.877057 | TA |
| **Indole-3-Propanoic Acid** | 190.123 | 7.652 | M+H | $C_{11}H_{11}NO_2$ | 4.2751E-6 | 0.198637 | TA |
| **Indole-3-Lactic Acid** | 188.075 | 4.038 | M+H-H2O | $C_{11}H_{11}NO_3$ | 7.1802E-4 | 0.558675 | TA |
| **Harmane** | 183.097 | 5.427 | M+H | $C_{12}H_{10}N_2$ | 8.4951E-4 | 0.565836 | TA |

**Table S3:** Significantly metabolic pathways in *T. asperellum* and *T. harzianum*

| No. | Pathway Name | -log (*p*) | Impact |
|---|---|---|---|
| **1** | **Phenylalanine, tyrosine, and tryptophan biosynthesis** | 2.564 | 0.0363 |
| **2** | **alpha-Linolenic acid metabolism** | 2.0114 | 0.0 |
| **3** | **Tryptophan metabolism** | 1.9377 | 0.31578 |
| **4** | **Arginine biosynthesis** | 1.6209 | 0.216 |
| **5** | **Ubiquinone and other terpenoid-quinone biosynthesis** | 1.5784 | 0.0 |
| **6** | **Sphingolipid metabolism** | 1.4294 | 0.06471 |
